# Intestine‐specific expression of human chimeric intestinal alkaline phosphatase attenuates Western diet‐induced barrier dysfunction and glucose intolerance

**DOI:** 10.14814/phy2.13790

**Published:** 2018-07-29

**Authors:** Siddhartha S. Ghosh, Hongliang He, Jing Wang, William Korzun, Paul J. Yannie, Shobha Ghosh

**Affiliations:** ^1^ Department of Internal Medicine VCU Medical Center Richmond Virginia; ^2^ Department of Clinical and Laboratory Sciences VCU Medical Center Richmond Virginia; ^3^ Hunter Homes McGuire VA Medical Center Richmond Virginia

**Keywords:** Glucose intolerance, Intestinal alkaline phosphatase, Intestinal barrier function, Western diet, zonulin

## Abstract

Intestinal epithelial cell derived alkaline phosphatase (IAP) dephosphorylates/detoxifies bacterial endotoxin lipopolysaccharide (LPS) in the gut lumen. We have earlier demonstrated that consumption of high‐fat high‐cholesterol containing western type‐diet (WD) significantly reduces IAP activity, increases intestinal permeability leading to increased plasma levels of LPS and glucose intolerance. Furthermore, oral supplementation with curcumin that increased IAP activity improved intestinal barrier function as well as glucose tolerance. To directly test the hypothesis that targeted increase in IAP would protect against WD‐induced metabolic consequences, we developed intestine‐specific IAP transgenic mice where expression of human chimeric IAP is under the control of intestine‐specific villin promoter. This chimeric human IAP contains domains from human IAP and human placental alkaline phosphatase, has a higher turnover number, narrower substrate specificity, and selectivity for bacterial LPS. Chimeric IAP was specifically and uniformly overexpressed in these IAP transgenic (IAPTg) mice along the entire length of the intestine. While IAP activity reduced from proximal P1 segment to distal P9 segment in wild‐type (WT) mice, this activity was maintained in the IAPTg mice. Dietary challenge with WD impaired glucose tolerance in WT mice and this intolerance was attenuated in IAPTg mice. Significant decrease in fecal zonulin, a marker for intestinal barrier dysfunction, in WD fed IAPTg mice and a corresponding decrease in translocation of orally administered nonabsorbable 4 kDa FITC dextran to plasma suggests that IAP overexpression improves intestinal barrier function. Thus, targeted increase in IAP activity represents a novel strategy to improve WD‐induced intestinal barrier dysfunction and glucose intolerance.

## Introduction

High‐fat and high‐cholesterol containing western type diet (WD)‐induced obesity remains one of the major causes for the development of metabolic syndrome and associated diseases such as type 2 diabetes (T2DM) and coronary artery disease. In addition to changes in lipid metabolism and excessive lipid accumulation, recent studies have also described direct effects of WD on gut microbiome and diet induced dysbiosis of gut flora is thought to contribute to the development of metabolic diseases (Sonnenburg and Sonnenburg 2014; Agus et al. 2016; Martinez et al. 2017). Extensive research is underway to define the changes in gut microbiome in several human diseases in order to develop correlation between gut dysbiosis and progress of these diseases (Selber‐Hnatiw et al. 2017). However, how does this diet‐induced dysbiosis “communicates” with the host and brings about the systemic metabolic changes is not completely defined. Under physiological conditions, an intact intestinal lining not only protects the host from direct interaction with pathogenic gut bacteria (likely to increase during dysbiosis) but also prevents the translocation of bacteria and bacterial endotoxin (e.g., Lipopolysaccharide, LPS) to systemic circulation. Altered gut mucosa with increased intestinal permeability is associated with several chronic inflammatory diseases and strong association between circulating gut bacteria‐derived LPS and metabolic diseases (such as T2DM and atherosclerosis) has shifted the focus from WD‐induced changes in gut microbiota *per se* to release of gut bacteria‐derived products (e.g., LPS) into circulation as the possible mechanism for the chronic inflammatory state underlying the development of these diseases (Nakarai et al. 2012). We demonstrated disruption of intestinal barrier function by WD resulting in significant release of bacterial LPS into the circulation of WD‐fed mice (Ghosh et al. 2014). Selective decontamination of the gut or oral supplementation with curcumin significantly improved the intestinal barrier function in WD fed LDLR‐/‐ mice resulting in decreased translocation of gut‐derived LPS into systemic circulation (Ghosh et al. 2014). This led to improved glucose tolerance and attenuated atherosclerosis without affecting plasma lipid profiles providing direct evidence that intestine is likely the “first line of defense” against the metabolic consequences of WD and targeted improvement of intestinal barrier function may represent a novel strategy to attenuate WD‐induced metabolic diseases.

Intestinal barrier consists of multiple layers including: (1) luminal intestinal alkaline phosphatase (IAP) that dephosphorylates LPS to detoxify it; (2) the mucus layer that provides a physical barrier preventing interactions between gut bacteria and intestinal epithelial cells; (3) the tight junctions between the epithelial cells that limit the paracellular transport of bacteria and/or bacterial products to systemic circulation; and (4) the antibacterial proteins secreted by the intestinal epithelial cells. The identity of the component(s) of intestinal barrier affected by WD or WD‐induced gut dysbiosis remains currently undefined. Furthermore, whether targeted improvement of *only* one layer of the intestinal barrier would be sufficient to prevent the translocation of bacterial‐derived products to systemic circulation has not been established.

In earlier studies, we demonstrated significant increase in IAP activity following oral supplementation with curcumin that also reduced WD‐induced glucose intolerance and atherosclerosis pointing to an important role for this first layer of the intestinal barrier (Ghosh et al. 2014). Intestinal alkaline phosphatase (IAP) is part of the luminal first line of defense and catalyzes the removal of one of the two phosphate groups from the toxic lipid A moiety of LPS producing monophosphoryl‐LPS that still binds to TLR4 but predominantly acts as an TLR4 antagonist (Bentala et al. 2002). Furthermore, Tan et al. (2015) have recently described the inability of monophosphoryl‐LPS to appropriately bind to CD14 to facilitate TLR4 endocytosis and downstream signaling. Therefore, IAP is central in maintaining the critical homeostasis that exists between the host and the luminal microbial environment (Bates et al. 2007) underscoring the importance of exploring supplementation with exogenous IAP to alleviate pathological conditions where host/microbial homeostasis is perturbed such as colitis or chronic inflammation. Consistently, Tuin et al. (2009) have reported a decrease in IAP in patients with inflammatory bowel disease and heat stable, chimeric human alkaline phosphatase is currently being evaluated as a protein therapeutic for gut dysbiosis, inflammatory bowel disease, and acute kidney injury (Kiffer‐Moreira et al. 2014). However, IAP is an acid‐labile enzyme that completely loses its catalytic activity below pH 5.0 (Golotin et al. 2015) precluding effective oral administration of an *active* enzyme although some beneficial effects of oral administration of IAP (purified calf intestinal alkaline phosphatase) to mice in drinking water have been reported (Ramasamy et al. 2011; Kaliannan et al. 2013). To directly test the hypothesis that targeted increase in IAP would protect against WD‐induced metabolic consequences, we developed intestine‐specific IAP transgenic mice expressing chimeric human IAP (developed by Kiffer‐Moreira et al., 2014). This chimeric human IAP contains domains from human IAP and human placental alkaline phosphatase, displays greatly increased heat stability, increased Zn^2+^ binding affinity, increased transphosphorylation, a higher turnover number, narrower substrate specificity, and selectivity for bacterial LPS. Significant improvement of WD‐induced glucose intolerance was noted in intestine‐specific IAP transgenic mice.

## Materials and Methods

### Production and screening of transgenic mice

All animal protocols were approved by the Institutional Animal Care and Use Committee at the Virginia Commonwealth University.

DNA containing the open reading frame of the heat stable, chimeric human intestinal alkaline phosphatase (IAP) containing the catalytic domain of human placental alkaline phosphatase that imparts enhanced catalytic efficiency (Kiffer‐Moreira et al. 2014) was synthesized by Invitrogen. A C‐terminal c‐myc tag was added along with 5’ Xma I and 3’ Kpn I sites and the required Kozak box and in‐frame start codon at the 5’ end. This synthetic sequence was cloned into pUC19 shuttle vector and the Xma I/Kpn I fragment was subsequently cloned into Villin promoter driven transgenic expression vector (12.4kbVillin‐ΔATG, Plasmid #19358) obtained from Addgene. The sequence of the chimeric transgene was confirmed by sequencing in both directions. The villin‐IAP chimeric transgene was separated from the vector sequence by digestion with Pme I, purified by agarose gel electrophoresis, and 15.5 kb fragment was injected into the pronuclei of fertilized mouse eggs obtained from super‐ovulated female mice (C57BL/6 or Balb‐c/C57BL/6 hybrids). The injected eggs were surgically transferred to oviducts of surrogate females. Offsprings were screened for integration of the transgene by PCR amplification of the tail DNA with the upstream primer sequence 5′‐CAGACACTCTATGCCTGTGTGGAG‐3′ and the downstream primer sequence 5′‐GGAACCTTACTTCTGTGGTGTGAC‐3′ to yield a 503‐bp product. The transgenic founder lines were maintained and propagated. Following initial characterization, automated genotyping was performed by Transnetyx, Inc. Transgenic (IAPTg) or nontransgenic (WT – wild‐type) littermates of both sexes were used for all the studies and the number as well as sex of mice included was therefore dependent on the litter size.

### Measurement of transgenic IAP expression

Nontransgenic (WT) and IAPTg littermates were euthanized and liver, kidney as well as various regions of the gastrointestinal tract were harvested. Tissues were homogenized in RIPA buffer and total extracts were subjected to SDS‐PAGE followed by western blot analyses. The presence of c‐myc tag on transgenic IAP protein was identified using rabbit polyclonal anti‐cmyc primary antibody (Sigma‐Aldrich, C3956). Total RNA was prepared using RNeasy Kit (Qiagen) and specific expression of transgenic IAP in different tissues was monitored by SYBR‐based real‐time PCR using 5’ primer spanning the Kozak box and start codon (5’‐CGCCACATGCAGGGACC‐3’) and 3’ primer spanning the c‐myc tag (5’‐CCGAAATCAGCTTCTGCTCGTCGG‐3’). A standard curve was generated using serial dilution of plasmid DNA and IAP copy number per μg total RNA in different tissues was calculated as described earlier (Zhao et al. 2007). Taqman gene expression assays from Applied Biosystems were used to determine the mRNA levels of endogenous IAP isoforms: AKP2 (Mm00475834_m1), AKP3 (Mm00475847_g1), AKP5 (Mm00475856_g1) and AKP6 (Mm01285814_g1).

### Measurement of IAP enzyme activity

The complete gastrointestinal tract starting at the base of the stomach to the tip of cecum was harvested. After collecting duodenum and jejunum, the remaining length of the intestine was cut into 9 equal segments. Tissues were homogenized in 50 mM Tris‐HCl buffer pH 7.5 containing 250 mmol/L sucrose. 0.2% Triton X‐100 (v/v) and protease inhibitors. The homogenates were centrifuged at 10,000 x g for 10 min and postmitochondrial supernatant was used to determine IAP activity as described earlier (de La Serre et al. 2010) using p‐nitrophenylphosphate as substrate and expressed as mmoles/h/mg total protein. Three different protein concentrations and two time points were used to ensure linearity.

### Intraperitoneal glucose tolerance tests

Nontransgenic (WT) or IAPTg mice (littermates) at 10 weeks of age were fed a high‐fat, high‐cholesterol western type diet (WD, TD88137, Harlan Teklad) which contained 21% fat, 0.15% cholesterol, and 19.5% casein by weight with no sodium cholate for 16 weeks. After an overnight fast, a single bolus of glucose (2 mg/g body weight) was given intraperitoneally. Blood glucose levels were determined by commercially available glucometer using tail vein blood at 0, 15, 30, 60, 120 min.

### Plasma analyses

Mice were fasted overnight and at the time of necropsy, blood was collected by cardiac puncture and plasma was frozen at −80°C until analyzed. Total plasma cholesterol and triglyceride levels were determined using the Cobas c311 automated chemistry analyzer, with reagents, calibrators, and controls from Roche Diagnostics. Plasma LPS levels were determined using Pierce LAL Chromogenic Endotoxin Kit (Thermo Fisher) and IL‐6 levels were measured by Ready‐set‐go ELISA kit from eBiosciences. Aliquots of undiluted plasma were also used to determine total IAP activity as described above.

### Translocation of FITC Dextran in vivo

Mice were orally gavaged with FITC‐dextran 4 kDa (600 *μ*g/kg body weight) dissolved in deionized water and plasma was collected after 4 h. Using three aliquots of plasma in duplicate, FITC fluorescence (excitation 488 nm and emission 518 nm) was measured using Perkin Elmer Victor three plate reader and concentration of FITC in plasma determined using a standard curve and expressed as ng/*μ*L of plasma.

### Fecal analyses

Feces were collected over a period of 24 h from mice fed WD for 16 weeks. Following vacuum drying in a dessicator, dried feces was powdered using glass pestle and mortar and the powder stored at −20°C until analyzed. Fecal analyses were performed by Salveo Diagnostics laboratory on a fee for service basis. For determining fecal IAP activity, dried feces was homogenized in water, centrifuged and the supernatant used for IAP activity measurement.

### FACS analyses of intestinal macrophages

At the time of euthanasia, one inch pieces of ileum and colon were collected, cleaned of any luminal contents and ~50 mg tissue piece was enzymatically digested to prepare single cell suspension as described earlier (Bie et al. 2011). Isolated cells were re‐suspended in FACS buffer containing Fc block and incubated with fluorescently labeled antibodies for 20 min at 4°C. After washing, 50,000 FITC‐labeled counting beads were added to each sample and analyzed by flow cytometry (BD Biosciences, Canto II) with stopping gate set to count 10,000 beads. The data were analyzed using FlowJO (Tree Star Inc.) software. The following antibodies were used: Antimouse CD45‐PE (leukocytes), antimouse CD11b‐PerCP‐Cy5.5 (macrophages) antimouse Ly6C‐APC and the respective isotype controls (all antibodies were obtained from eBiosciences).

### Statistical analyses

Comparison between two groups was performed using Student's t‐test and *P* < 0.05 was considered significant. One‐way ANOVA with Bonferroni post hoc correction was used for multiple comparisons and details are included in Figure legends.

## Results

### Characterization of IAPTg mice

**Figure 1:**
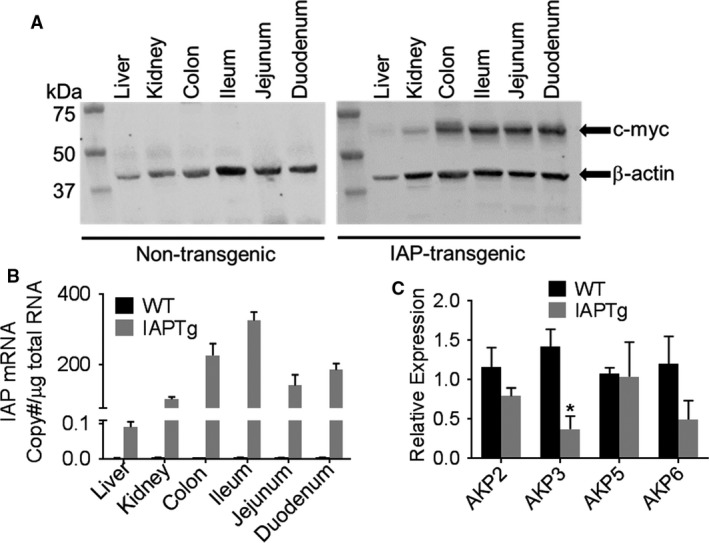
Villin promoter‐driven intestine‐specific expression of IAP. At 10 weeks of age nontransgneic (WT) and IAPTg littermates were euthanized and RNA as well as total protein extracts were prepared from the indicated tissues. (A) Total protein extracts from indicated tissues were analyzed by Western blot analyses using primary antibody to c‐myc tag and β‐actin. Representative blots are shown and immune‐reactive bands are indicated by arrows. (B) mRNA levels of chimeric IAP were quantified by QPCR as described under Methods and IAP mRNA copy number was calculated. (C) Total RNA from ileum was used to determine the mRNA levels of indicated endogenous IAP isoforms by QPCR. Data are expressed as Mean ± SD,* n* = 3. *P < 0.05.

IAPTg mice were identified by PCR amplification of tail DNA. One founder line in C57BL/6 background and one in Balb‐c/C57BL/6 hybrid background were obtained. Both transgenic founder lines were analyzed for tissue distribution of IAP. Similar expression profiles were obtained with both lines, and data for the C57BL/6 founder line is shown in Figure 1. While some expression of IAP transgene, as identified by c‐myc tag, was seen in liver and kidney, significantly higher expression was seen in the duodenum, jejunum, ileum, and colon (Fig. 1A) consistent with the reported use of this Villin promoter construct (Madison et al. 2002). In accordance with the observed protein expression, higher transgenic IAP mRNA levels were observed in tissues from IAPTg mice compared to nontransgenic (WT) mice (Fig. 1B). These data confirm the successful development of intestine‐specific IAP transgenic mice. Genetic deletion of the major intestinal isoform, AKP3, led to a compensatory increase in the expression of other isoform (AKP6) resulting in no apparent change in total IAP activity (Narisawa et al. 2007). To evaluate the effects of transgenic overexpression of IAP on the expression of endogenous isoforms, mRNA levels of AKP2, AKP3, AKP5, and AKP6 were determined by QPCR. While no significant difference was noted in the expression of AKP2, AKP5, and AKP6, expression of AKP3 was significantly reduced (Fig. 1C).

**Figure 2:**
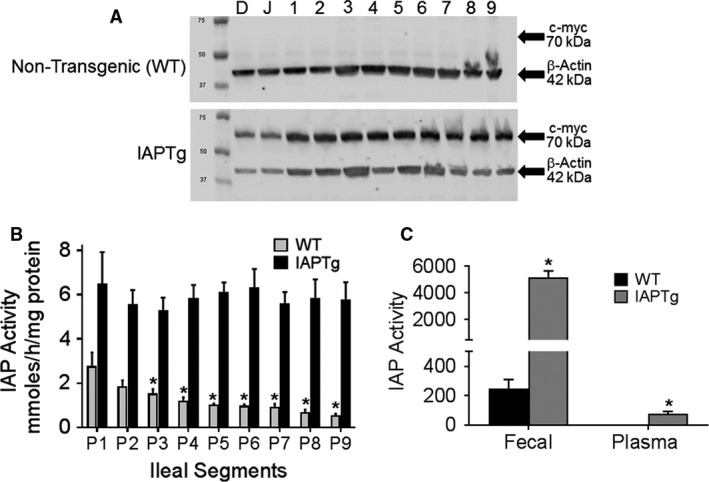
IAP is uniformly expressed along the entire length of the ileum. (A) Duodenum (D), Jejunum (J) and 1–9 equal segments of ileum from jejunum to colon were harvested from nontransgenic (WT) and IAPTg littermates. Total protein extracts were analyzed by Western blot analyses and representative blots are shown. Immunoreactive bands for C‐terminal c‐myc tag and β‐actin are indicated by arrows. (B) Ileal segments P1 through P9 harvested from nontransgenic (WT) and IAPTg littermates were used to assay for IAP enzyme activity as described under Methods. Data are presented as Mean ± SD,* n* = 3. *P < 0.05 compared to P1 segment. (C) Fecal and plasma IAP activity was determined as described under “Method” and data are presented as Mean ± SD,* n* = 4 for each genotype. *P < 0.05.

Variations exist in the expression as well as activity of IAP along the length of the ileum (Bilski et al. 2017) and therefore, expression of IAP transgene was also monitored along the entire length of the ileum. Almost uniform protein expression of c‐myc tagged IAP transgene was noted along the ileum (Fig. 2A) and correspondingly no significant difference in IAP enzyme activity was seen among the nine segments of ileum (Fig. 2B). In contrast, there was a gradual and significant decrease in IAP activity in nontransgenic WT mice from proximal segment P1 to distal segment P9. To confirm secretion of IAP into the intestinal lumen, fecal IAP activity was measured. As shown in Figure 2C, significantly higher IAP activity was present in feces from IAPTg mice (5181.67 ± 942.53 vs. 246.46 ± 128.17, *P* < 0.0001). Plasma IAP activity, on the other hand was much lower than fecal activity in WT as well as IAPTg mice.

Since there was no significant difference in IAP expression between the 2 founder lines tested, the line in pure C57BL/6 background was chosen for further studies.

### Transgenic expression of IAP improves WD‐induced glucose intolerance

**Figure 3:**
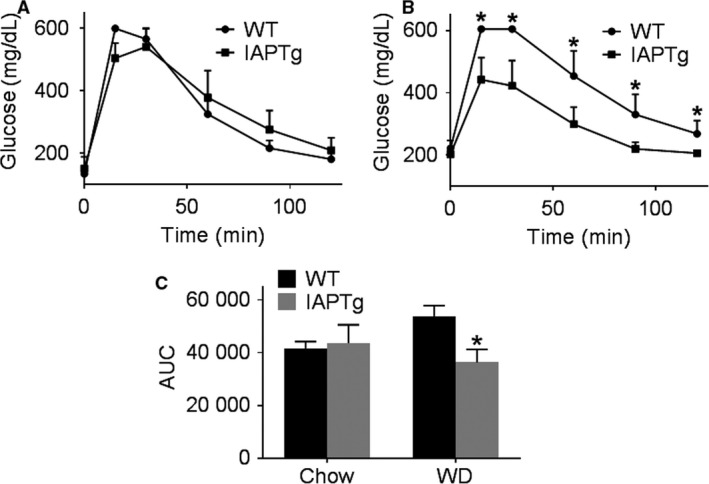
Transgenic expression of IAP improves WD‐induced glucose intolerance. Nontransgenic wild‐type (WT) and IAPTg littermates of both sexes were either fed chow or WD for 16 weeks. After an overnight fast, intraperitoneal glucose tolerance tests were performed. Blood glucose levels over time for chow fed (A) or WD fed (B) mice are shown (Mean ± SD,* n* = 6). (C) Area under the curve (AUC) for the plots for individual mice included in the mean data shown in (A and B) were determined and Mean ± SD,* n* = 6 of AUC are shown. *P < 0.05.

Based on our earlier observation that attenuation of WD‐induced glucose intolerance by oral supplementation with curcumin was associated with increase in IAP activity (Ghosh et al. 2014), intestine‐specific IAPTg mice were developed to directly test this causal relationship. Although no significant difference in blood glucose clearance was noted between WT and IAPTg mice on chow diet (Fig. 3A), WD feeding for 16 weeks led to significant intolerance in WT mice (Fig. 3B and C, note the significant difference in AUC). Consistent with increased expression and activity of IAP in the intestine of IAPTg mice, WD‐induced glucose intolerance was significantly attenuated in these mice.

**Figure 4:**
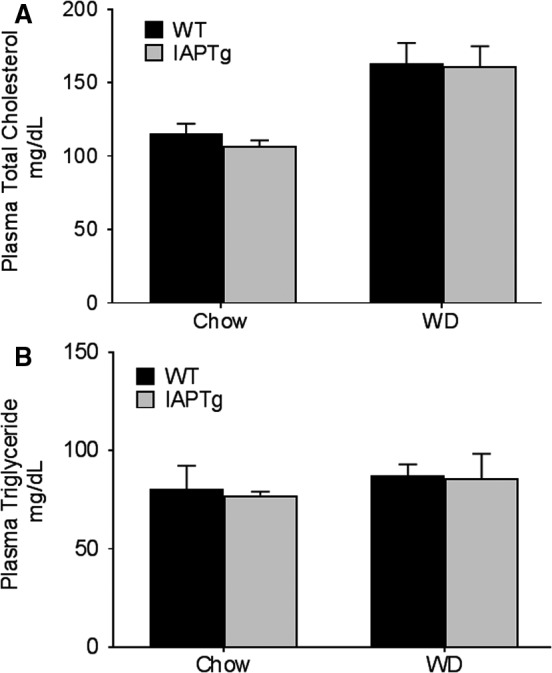
Transgenic expression of IAP does not significantly alter plasma lipid profiles: Nontransgenic wild‐type (WT) and IAPTg littermates of both sexes were either fed chow or WD for 16 weeks. After an overnight fast, mice were euthanized and blood was collected by cardiac puncture. Plasma total cholesterol (A) and triglycerides (B) were determined as described under methods. Data (Mean ± SD,* n* = 6) are shown.

WD‐induced changes in plasma lipids are thought to underlie downstream metabolic changes and to examine whether transgenic expression of IAP dependent changes in plasma lipid levels underlie the observed improvement of glucose tolerance, total plasma cholesterol, and triglyceride levels were monitored. As shown in Figure 4A, whereas there was a diet‐dependent increase in plasma cholesterol levels, there was no difference between the two genotypes either on chow diet or WD. WD feeding did not significantly affect the plasma triglyceride levels in either genotype. These data indicate that intestine‐specific transgenic expression of IAP does not affect plasma lipid levels. It should be noted that these mice are on C57BL/6 background and not on a hyperlipidemic (LDLR‐/‐ or ApoE‐/‐) background.

### Transgenic expression of IAP improved intestinal barrier function

**Figure 5:**
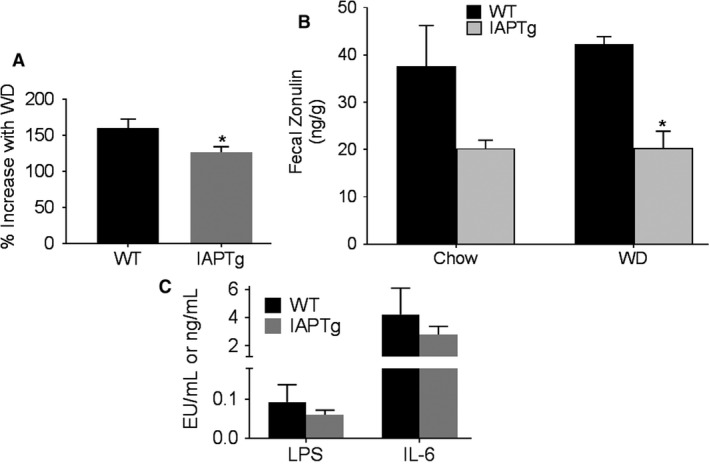
Transgenic expression of IAP improves intestinal barrier function: Nontransgenic wild‐type (WT) and IAPTg littermates of both sexes were either fed chow or WD for 16 weeks. (A) After an overnight fast, mice were gavaged with FITC‐dextran and euthanized after 4 h. Plasma FITC levels were measured as described under methods. Data (Mean ± SD,* n* = 6) are presented as percent increase in plasma FITC levels by WD feeding. (B) Feces from individual mice were collected over a period of 48 h and fecal zonulin levels were determined. Data (Mean ± SD,* n* = 6) are presented as ng/g of dried feces. (C) Plasma LPS (EU/mL) and IL‐6 (ng/mL) levels were determined as described under “Methods” and Data are presented as Mean ± SD,* n* = 4 for each genotype. *P < 0.05.

We have earlier demonstrated disruption of WD‐induced intestinal barrier function and proposed its causal role in the development of WD‐induced metabolic changes. The effects of IAP over expression in the ileum on changes in intestinal permeability as a result of WD feeding were directly evaluated by monitoring the translocation of orally administered 4 kDa FITC‐dextran. WD feeding led to >50% increase in plasma FITC levels but this increase was significantly reduced in IAPTg mice (Fig. 5A) demonstrating an improvement in intestinal permeability.

Inappropriate upregulation of zonulin secretion from the intestinal epithelial cells into the lumen increases paracellular permeability (Fasano et al. 2000; El Asmar et al. 2002) and fecal levels of Zonulin are used as a clinical measure of intestinal permeability. Therefore, fecal zonulin levels were monitored and as shown in Figure 5B, compared to WT mice, reduced levels of fecal zonulin was seen in IAPTg mice although this decrease reached statistical significance only in the WD fed group (likely due to greater variation in chow fed WT group). Nonetheless, taken together these data indicate improved intestinal barrier function in IAPTg mice. Consistently, plasma LPS as well as IL‐6 levels were lower in WD‐fed IAPTg compared to WT controls although these differences did not reach statistical significance (Fig. 5C).

### Transgenic expression of IAP improved intestinal inflammation

**Figure 6:**
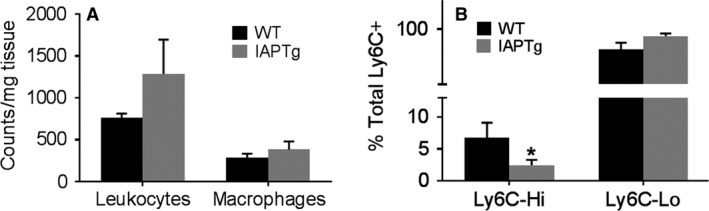
Transgenic expression of IAP improves intestinal inflammation: Nontransgenic wild‐type (WT) and IAPTg littermates of both sexes were fed WD for 16 weeks. Single cell preparations of ileum were prepared and stained for leukocytes and macrophages as described under “Methods” and analyzed by flow cytometry. (A) Total number of CD45 +  leukocytes and CD11b+ macrophages per mg of tissue are shown (Mean ± SD,* n* = 6). (B) Percentage of Ly6C‐Hi and Ly6C‐Lo within the total CD45 + CD11b+Ly6C+ population was determined and data are presented as Mean ± SD,* n* = 6. *P < 0.05.

Disruption of the different layers of the intestinal barrier leads to increase in systemic as well as intestinal inflammation. To assess the effects of increased IAP expression on intestinal inflammation, the number and phenotype of intestinal macrophages was evaluated. As shown in Figure 6, while there was no significant difference in the number of total leukocytes or macrophages in ileum (Panel A), significantly lower percentage of macrophages were Ly6C‐Hi or polarized toward a proinflammatory phenotype in IAPTg mice (Panel B) suggestive of lower intestinal inflammation in these mice.

## Discussion

On the basis of the observed WD‐induced reduction in intestinal IAP and subsequent increase in intestinal permeability leading to glucose intolerance, in this study we sought to examine the effects of intestine‐specific transgenic overexpression of IAP. We report the successful development of intestine‐specific IAP transgenic mice with uniform expression and activity of human chimeric IAP along the intestine. Significant increase in fecal IAP activity confirmed the secretion of IAP into the lumen of the intestine. This targeted increase in IAP activity led to improved intestinal barrier function and attenuation of WD‐induced increase in intestinal permeability. More importantly, WD‐induced glucose intolerance was significantly attenuated in IAPTg mice providing direct evidence for a causal role of WD‐induced reduction in intestinal IAP in the development of glucose intolerance.

While effects of WD on the induction of gut dysbiosis are extensively investigated, recent studies including the ones from our laboratory, have demonstrated the causative role of WD‐induced increase in intestinal permeability and resulting increase in the translocation of luminal LPS (or endotoxemia) in the development of metabolic diseases. WD not only causes local intestinal inflammation (Ding et al. 2010) but excess chylomicron formation during WD feeding facilitates the translocation of luminal LPS, internalized by the intestinal epithelial cells from the apical side, to systemic circulation (Ghoshal et al. 2009). This local and systemic inflammation lead to overexpression of proinflammatory cytokines (Park et al. 2007) that increase gut permeability (Bruewer et al. 2003) and further increase in LPS translocation (Cani et al. 2008) leading to a vicious cycle of metabolic endotoxemia that leads to chronic inflammatory state critical to the development of metabolic diseases (Moreira et al. 2012). Consistently, patients with obesity, diabetes, CVD, and NAFLD have higher circulating LPS levels than healthy individuals (Creely et al. 2007; Thuy et al. 2008; Pussinen et al. 2011) These observations underscore the importance of improving intestinal barrier function and/or reducing the levels of active LPS. IAP, secreted by the intestinal epithelial cells, is appropriately positioned to facilitate both these processes. In addition to dephosphorylation of LPS, IAP is also thought to attenuate inflammatory signaling by other bacterial components such as CpG DNA and flagellin without direct effects on bacteria (Chen et al. 2010). Consistently, reduced polarization of intestinal macrophages to proinflammatory Ly6C‐Hi phenotype was seen in IAPTg mice. Furthermore, the observed reduction in FITC‐dextran translocation seen in IAPTg mice demonstrates that intestine‐specific transgenic expression of human chimeric IAP indeed improves intestinal barrier function establishing the causal link between IAP expression and intestinal permeability.

Intestinal barrier function and paracellular transport is maintained/regulated by appropriate expression and organization of tight junctions consisting of multiple proteins (e.g., membrane spanning proteins occludins or claudins and other tight junction proteins such as ZO‐1, ZO‐2, and ZO‐3) as well as organization/contractility of the actin microfilaments (Anderson 2001; Tsukita et al. 2001). Furthermore, these tight junctions open and close in response to a variety of stimuli including dietary components and luminal microbial factors. One such pathway that is relevant to intestinal barrier dysfunction and the disease process is the zonulin pathway. Analogous to bacterial toxin (zonula occludens), inappropriate upregulation of zonulin secretion from the intestinal epithelial cells into the lumen increases paracellular permeability (Fasano et al. 2000; El Asmar et al. 2002) and fecal levels of Zonulin are used as a clinical measure of intestinal permeability. Data presented herein show that transgenic expression of IAP attenuates WD‐induced increase in fecal zonulin providing another likely mechanism by which IAP could modulates intestinal barrier function in addition to reduces luminal LPS or LPS‐mediated inflammatory changes in the intestinal epithelial cell layer. While certain bacteria (El Asmar et al. 2002) and gliadin (Clemente et al. 2003) are recognized as the two major triggers for zonulin release, the mechanism by which IAP affects the zonulin pathway remains to be established.

While the causal role of the disruption of intestinal barrier function in the development of inflammatory bowel disease is well established (Lee et al. 2018), its role is also being causally associated with the development of multiple diseases including diabetes, NAFLD (Wang et al. 2018), COPD (Sprooten et al. 2018), Parkinson's disease (Schwiertz et al. 2018), multiple sclerosis (Buscarinu et al. 2018). Data presented herein show that transgenic expression of IAP led to improved intestinal barrier function as well as attenuation of WD‐induced glucose intolerance. These data are not only consistent with the fact that mice deficient in IAP develop Type 2 diabetes (Kaliannan et al. 2013) and reduced levels of fecal IAP is seen in diabetic subjects (Lassenius et al. 2017) but also with our earlier demonstration of improved glucose tolerance by oral supplementation with curcumin that increased IAP activity (Ghosh et al. 2014). It is also noteworthy that other interventions such as berberine supplementation also improved the diabetic status by enhancing the intestinal barrier function (Shan et al. 2013) underscoring the importance of IAP as potential therapeutic strategy to improve intestinal barrier function.

Exogenous supplementation with IAP thus represents a viable strategy for decreasing the development of diseases linked to increase in intestinal permeability and systemic translocation of bacteria/bacterial products. Instability and loss of activity of IAP at pH < 5.0, precludes its oral administration without specific enteric formulation to circumvent gastric inactivation. Consistently, Tuin et al. (2009) reported attenuation of DSS‐induced colitis in rats by supplementation with IAP given as enteric‐coated tablets that prevents dissolution in the stomach and inactivation of acid labile IAP. To bypass gastric inactivation, Lukas et al. (2010) administered 30,000U of alkaline phosphatase intraduodenally and reported successful treatment of patients with severe ulcerative colitis. The current study along with demonstrated success with administration of exogenous IAP pave the way for developing appropriate platforms for intestinal delivery of active IAP enzyme (withstanding the low gastric pH‐dependent inactivation/degradation) as a novel and simple/noninvasive strategy for attenuation of WD‐induced metabolic changes including glucose intolerance and diabetes.

## Conflict of Interest

None.

## References

[phy213790-bib-0001] Agus, A. , J. Denizot , J. Thévenot , M. Martinez‐Medina , S. Massier , P. Sauvanet , et al. 2016 Western diet induces a shift in microbiota composition enhancing susceptibility to Adherent‐Invasive E. coli infection and intestinal inflammation. Sci. Rep. 6:19032. 26742586 10.1038/srep19032 PMC4705701

[phy213790-bib-0002] Anderson, J. M. 2001 Molecular structure of tight junctions and their role in epithelial transport. News Physiol. Sci. 16:126–130. 11443232 10.1152/physiologyonline.2001.16.3.126

[phy213790-bib-0003] Bates, J. , J. Akerlund , E. Mittge , and K. Guillemin . 2007 Intestinal alkaline phosphatase detoxifies lipopolysaccharide and prevents inflammation in zebrafish in response to gut microbiota. Cell Host Microbe 2:371–382. 18078689 10.1016/j.chom.2007.10.010 PMC2730374

[phy213790-bib-0004] Bentala, H. , W. R. Verweij , A. Huizinga‐Van der Vlag , A. M. van Loenen‐Weemaes , D. K. Meijer , and K. Poelstra . 2002 Removal of phosphate from lipid A as a strategy to detoxify lipopolysaccharide. Shock. 18:561–566. 12462566 10.1097/00024382-200212000-00013

[phy213790-bib-0005] Bie, J. , B. Zhao , and S. Ghosh . 2011 Atherosclerotic lesion progression is attenuated by reconstitution with bone marrow from macrophage‐specific cholesteryl ester hydrolase transgenic mice. Am. J. Physiol. Regul. Integr. Comp. Physiol. 301:R967–R974. 21795638 10.1152/ajpregu.00277.2011 PMC3197345

[phy213790-bib-0006] Bilski, J. , A. Mazur‐Bialy , D. Wojcik , J. Zahradnik‐Bilska , B. Brzozowski , M. Magierowski , et al. 2017 The role of intestinal alkaline phosphatase in inflammatory disorders of gastrointestinal tract. Mediators Inflamm. 2017:9074601. 28316376 10.1155/2017/9074601 PMC5339520

[phy213790-bib-0007] Bruewer, M. , A. Luegering , T. Kucharzik , C. A. Parkos , J. L. Madara , A. M. Hopkins , et al. 2003 Proinflammatory cytokines disrupt epithelial barrier function by apoptosis‐independent mechanisms. J. Immunol. 171:6164–6172. 14634132 10.4049/jimmunol.171.11.6164

[phy213790-bib-0008] Buscarinu, M. C. , S. Romano , R. Mechelli , R. Pizzolato Umeton , M. Ferraldeschi , A. Fornasiero , et al. 2018 Intestinal permeability in relapsing‐remitting multiple sclerosis. Neurotherapeutics 15:68–74. 29119385 10.1007/s13311-017-0582-3 PMC5794695

[phy213790-bib-0009] Cani, P. D. , R. Bibiloni , C. Knauf , A. Waget , A. M. Neyrinck , N. M. Delzenne , et al. 2008 Changes in gut microbiota control metabolic endotoxemia‐induced inflammation in high‐fat diet‐induced obesity and diabetes in mice. Diabetes 57:1470–1481. 18305141 10.2337/db07-1403

[phy213790-bib-0010] Chen, K. T. , M. S. Malo , A. K. Moss , S. Zeller , P. Johnson , F. Ebrahimi , et al. 2010 Identification of specific targets for the gut mucosal defense factor intestinal alkaline phosphatase. Am. J. Physiol. Gastrointest. Liver Physiol. 299:G467–G475. 20489044 10.1152/ajpgi.00364.2009 PMC2928538

[phy213790-bib-0011] Clemente, M. G. , S. De Virgiliis , J. S. Kang , R. Macatagney , M. P. Musu , M. R. Di Pierro , et al. 2003 Early effects of gliadin on enterocyte intracellular signalling involved in intestinal barrier function. Gut 52:218–223. 12524403 10.1136/gut.52.2.218 PMC1774976

[phy213790-bib-0012] Creely, S. J. , P. G. McTernan , C. M. Kusminski , F. M. Fisher , N. F. Da Silva , M. Khanolkar , et al. 2007 Lipopolysaccharide activates an innate immune system response in human adipose tissue in obesity and type 2 diabetes. Am. J. Physiol. Endocrinol. Metab. 292:E740–E747. 17090751 10.1152/ajpendo.00302.2006

[phy213790-bib-0013] Ding, S. , M. M. Chi , B. P. Scull , R. Rigby , N. M. Schwerbrock , S. Magness , et al. 2010 High‐fat diet: bacteria interactions promote intestinal inflammation which precedes and correlates with obesity and insulin resistance in mouse. PLoS ONE 5:e12191. 20808947 10.1371/journal.pone.0012191 PMC2922379

[phy213790-bib-0014] El Asmar, R. , P. Panigrahi , P. Bamford , I. Berti , T. Not , G. V. Coppa , et al. 2002 Host‐dependent zonulin secretion causes the impairment of the small intestine barrier function after bacterial exposure. Gastroenterology 123:1607–1615. 12404235 10.1053/gast.2002.36578

[phy213790-bib-0015] Fasano, A. , T. Not , W. Wang , S. Uzzau , I. Berti , A. Tommasini , et al. 2000 Zonulin, a newly discovered modulator of intestinal permeability, and its expression in coeliac disease. Lancet 355:1518–1519. 10801176 10.1016/S0140-6736(00)02169-3

[phy213790-bib-0016] Ghosh, S. S. , J. Bie , J. Wang , and S. Ghosh . 2014 Oral supplementation with non‐absorbable antibiotics or curcumin attenuates western diet‐induced atherosclerosis and glucose intolerance in LDLR‐/‐ mice–role of intestinal permeability and macrophage activation. PLoS ONE 9:e108577. 25251395 10.1371/journal.pone.0108577 PMC4177397

[phy213790-bib-0017] Ghoshal, S. , J. Witta , J. Zhong , W. de Villiers , and E. Eckhardt . 2009 Chylomicrons promote intestinal absorption of lipopolysaccharides. J. Lipid Res. 50:90–97. 18815435 10.1194/jlr.M800156-JLR200

[phy213790-bib-0018] Golotin, V. , L. Balabanova , G. Likhatskaya , and V. Rasskazov . 2015 Recombinant production and characterization of a highly active alkaline phosphatase from marine bacterium cobetia marina. Mar Biotechnol (NY). 17:130–143. 25260971 10.1007/s10126-014-9601-0

[phy213790-bib-0019] Kaliannan, K. , S. R. Hamarneh , K. P. Economopoulos , S. Nasrin Alam , O. Moaven , P. Patel , et al. 2013 Intestinal alkaline phosphatase prevents metabolic syndrome in mice. Proc. Natl. Acad. Sci. USA. 110:7003–7008. 23569246 10.1073/pnas.1220180110 PMC3637741

[phy213790-bib-0020] Kiffer‐Moreira, T. , C. R. Sheen , K. C. Gasque , M. Bolean , P. Ciancaglini , A. van Elsas , et al. 2014 Catalytic signature of a heat‐stable, chimeric human alkaline phosphatase with therapeutic potential. PLoS ONE 9:e89374. 24586729 10.1371/journal.pone.0089374 PMC3933536

[phy213790-bib-0021] de La Serre, C. B. , C. L. Ellis , J. Lee , A. L. Hartman , and J. C. Rutledge . 2010 Propensity to high‐fat diet‐induced obesity in rats is associated with changes in the gut microbiota and gut inflammation. Am. J. Physiol. Gastrointest. Liver Physiol. 299:G440–G448. 20508158 10.1152/ajpgi.00098.2010 PMC2928532

[phy213790-bib-0022] Lassenius, M. I. , C. L. Fogarty , M. Blaut , K. Haimila , L. Riittinen , A. Paju , et al. FinnDiane Study Group . 2017 Intestinal alkaline phosphatase at the crossroad of intestinal health and disease ‐ a putative role in type 1 diabetes. J. Intern. Med. 281:586–600. 28393441 10.1111/joim.12607

[phy213790-bib-0023] Lee, J. Y. , V. C. Wasinger , Y. Y. Yau , E. Chuang , V. Yajnik , and R. W. Leong . 2018 Molecular pathophysiology of epithelial barrier dysfunction in inflammatory bowel diseases. Proteomes. 6: pii E17. 29614738 10.3390/proteomes6020017 PMC6027334

[phy213790-bib-0024] Lukas, M. , P. Drastich , M. Konecny , P. Gionchetti , O. Urban , F. Cantoni , et al. 2010 Exogenous alkaline phosphatase for the treatment of patients with moderate to severe ulcerative colitis. Inflamm. Bowel Dis. 16:1180–1186. 19885903 10.1002/ibd.21161

[phy213790-bib-0025] Madison, B. B. , L. Dunbar , X. T. Qiao , K. Braunstein , E. Braunstein , and D. L. Gumucio . 2002 Cis elements of the villin gene control expression in restricted domains of the vertical (crypt) and horizontal (duodenum, cecum) axes of the intestine. J. Biol. Chem. 277:33275–33283. 12065599 10.1074/jbc.M204935200

[phy213790-bib-0026] Martinez, K. B. , V. Leone , and E. B. Chang . 2017 Western diets, gut dysbiosis, and metabolic diseases: Are they linked? Gut. Microbes 8:130–142. 28059614 10.1080/19490976.2016.1270811 PMC5390820

[phy213790-bib-0027] Moreira, A. P. , T. F. Texeira , A. B. Ferreira , M. C. Peluzio , and R. C. G. Alfenas . 2012 Influence of a high‐fat diet on gut microbiota, intestinal permeability and metabolic endotoxaemia. Br. J. Nutr. 108:801–809. 22717075 10.1017/S0007114512001213

[phy213790-bib-0028] Nakarai, H. , A. Yamashita , S. Nagayasu , M. Iwashita , S. Kumamoto , H. Ohyama , et al. 2012 Adipocyte‐macrophage interaction may mediate LPS‐induced low‐grade inflammation: potential link with metabolic complications. Innate Immun. 18:164–170. 21239459 10.1177/1753425910393370

[phy213790-bib-0029] Narisawa, S. , M. F. Hoylaerts , K. S. Doctor , M. N. Fukuda , D. H. Alpers , and J. L. Millán . 2007 A novel phosphatase upregulated in Akp3 knockout mice. Am. J. Physiol. Gastrointest. Liver Physiol. 293:G1068–G1077. 17901166 10.1152/ajpgi.00073.2007

[phy213790-bib-0030] Park, E. J. , M. Suh , B. Thomson , D. W. Ma , K. Ramanujam , A. B. Thomson , et al. 2007 Dietary ganglioside inhibits acute inflammatory signals in intestinal mucosa and blood induced by systemic inflammation of *Escherichia coli* lipopolysaccharide. Shock. 28:112–117. 17510604 10.1097/SHK.0b013e3180310fec

[phy213790-bib-0031] Pussinen, P. J. , A. S. Havulinna , M. Lehto , J. Sundvall , and V. Salomaa . 2011 Endotoxemia is associated with an increased risk of incident diabetes. Diabetes Care 34:392–397. 21270197 10.2337/dc10-1676 PMC3024355

[phy213790-bib-0032] Ramasamy, S. , D. D. Nguyen , M. A. Eston , S. N. Alam , A. K. Moss , F. Ebrahimi , et al. 2011 Intestinal alkaline phosphatase has beneficial effects in mouse models of chronic colitis. Inflamm. Bowel Dis. 17:532–542. 20645323 10.1002/ibd.21377 PMC3154118

[phy213790-bib-0033] Schwiertz, A. , J. Spiegel , U. Dillmann , D. Grundmann , J. Bürmann , K. Faßbender , et al. 2018 Fecal markers of intestinal inflammation and intestinal permeability are elevated in Parkinson's disease. Parkinsonism Relat. Disord. 50:104–107. 29454662 10.1016/j.parkreldis.2018.02.022

[phy213790-bib-0034] Selber‐Hnatiw, S. , B. Rukundo , M. Ahmadi , H. Akoubi , H. Al‐Bizri , A. F. Aliu , et al. 2017 Human gut microbiota: toward an ecology of disease. Front. Microbiol. 8:1265. 28769880 10.3389/fmicb.2017.01265 PMC5511848

[phy213790-bib-0035] Shan, C. Y. , J. H. Yang , Y. Kong , X. Y. Wang , M. Y. Zheng , Y. G. Xu , et al. 2013 Alteration of the intestinal barrier and GLP2 secretion in Berberine‐treated type 2 diabetic rats. J. Endocrinol. 218:255–262. 23757509 10.1530/JOE-13-0184

[phy213790-bib-0036] Sonnenburg, E. D. , and J. L. Sonnenburg . 2014 Starving our microbial self: the deleterious consequences of a diet deficient in microbiota‐accessible carbohydrates. Cell Metab. 20:779–786. 25156449 10.1016/j.cmet.2014.07.003 PMC4896489

[phy213790-bib-0037] Sprooten, R. T. M. , K. Lenaerts , D. C. W. Braeken , I. Grimbergen , E. P. Rutten , E. F. M. Wouters , et al. 2018 Increased small intestinal permeability during severe acute exacerbations of COPD. Respiration 000:000–000. 10.1159/000485935. [Epub ahead of print] PMC5985742 29393240

[phy213790-bib-0038] Tan, Y. , I. Zanconi , T. W. Cullen , A. L. Goodman , and J. C. Cagan . 2015 Mechanisms of toll‐like receptor 4 endocytosis reveal a common immune‐evasion strategy used by pathogenic and commensal bacteria. Immunity 43:909–922. 26546281 10.1016/j.immuni.2015.10.008 PMC4685471

[phy213790-bib-0039] Thuy, S. , R. Ladurner , V. Volynets , S. Wagner , S. Strahl , A. Königsrainer , et al. 2008 Nonalcoholic fatty liver disease in humans is associated with increased plasma endotoxin and plasminogen activator inhibitor 1 concentrations and with fructose intake. J. Nutr. 138:1452–1455. 18641190 10.1093/jn/138.8.1452

[phy213790-bib-0040] Tsukita, S. , M. Furuse , and M. Itoh . 2001 Multifunctional strands in tight junctions. Nat. Rev. Mol. Cell Biol. 2:285–293. 11283726 10.1038/35067088

[phy213790-bib-0041] Tuin, A. , K. Poelstra , A. de Jager‐Krikken , L. Bok , W. Raaben , M. P. Velders , et al. 2009 Role of alkaline phosphatase in colitis in man and rats. Gut 58:379–387. 18852260 10.1136/gut.2007.128868

[phy213790-bib-0042] Wang, W. , J. Zhao , W. Gui , D. Sun , H. Dai , L. Xiao , et al. 2018 Tauroursodeoxycholic acid inhibits intestinal inflammation and barrier disruption in mice with non‐alcoholic fatty liver disease. Br. J. Pharmacol. 175:469–484. 29139555 10.1111/bph.14095 PMC5773980

[phy213790-bib-0043] Zhao, B. , J. Song , W. N. Chow , R. W. St Clair , L. L. Rudel , and S. Ghosh . 2007 Macrophage‐specific transgenic expression of cholesteryl ester hydrolase significantly reduces atherosclerosis and lesion necrosis in Ldlr mice. J. Clin. Invest. 117:2983–2992. 17885686 10.1172/JCI30485 PMC1978419

